# Sensing of double-stranded RNA in human cells: molecular mechanisms and cellular consequences

**DOI:** 10.1042/BST20250259

**Published:** 2026-03-24

**Authors:** Julia Cieslicka, Karolina Pianka, Karolina Drazkowska, Pawel J. Sikorski

**Affiliations:** Laboratory of Epitranscriptomics, Faculty of Biology, Biological and Chemical Research Centre, University of Warsaw, Warsaw, Poland

**Keywords:** antiviral response, double-stranded RNA, innate immune sensing, RNA modifications, self vs non-self discrimination

## Abstract

Double-stranded RNA (dsRNA) is a universal indicator of viral replication and dysregulated RNA metabolism. Detection of dsRNA triggers some of the most powerful innate immune responses in human cells. Although these molecules differ in origin and structure, viral dsRNAs share the defining geometric and electrostatic features of the A-form helix, enabling their sequence-independent recognition by multiple sensor systems. Cytosolic receptors, like retinoic acid-inducible gene I (RIG-I), melanoma differentiation associated gene 5 (MDA5), and protein kinase R (PKR), as well as the oligoadenylate synthase (OAS)/RNase L pathway, convert dsRNA binding into interferon induction, translational arrest, and widespread RNA decay, while endosomal Toll-like receptor 3 (TLR3) and the inflammasome sensor NLR family pyrin domain containing 1 (NLRP1) expand surveillance to internalised or structurally disruptive RNAs. Counterbalancing these pathways, the RNA-editing enzyme adenosine deaminase acting on RNA 1 (ADAR1) marks endogenous dsRNA through A-to-I conversion, preventing inadvertent activation of innate immune response and maintaining self versus non-self discrimination. Although all of these sensors recognise the A-form helix, each extracts distinct structural and chemical information from dsRNA and converts it into a specific response: RIG-I detects short duplexes with 5′-triphosphorylated ends; MDA5 assembles cooperatively along long uninterrupted helices; PKR integrates duplex length with translational control; OAS proteins act as strict reporters of helix regularity; and TLR3 as well as NLRP1 respond to dsRNA in compartment- and context-dependent ways. Epitranscriptomic marks and chemical modifications—including 2′-*O*-methylation, *N*^6^-methyladenosine, pseudouridine, and ADAR1-mediated inosine—further refine sensing by modulating helical stability and end structure, establishing a biochemical ‘self-code’ that shapes RNA immunogenicity. Together, these pathways form an integrated network that distinguishes between viral and endogenous dsRNA and coordinates antiviral defence with immune tolerance.

## Introduction

**Figure 1:**
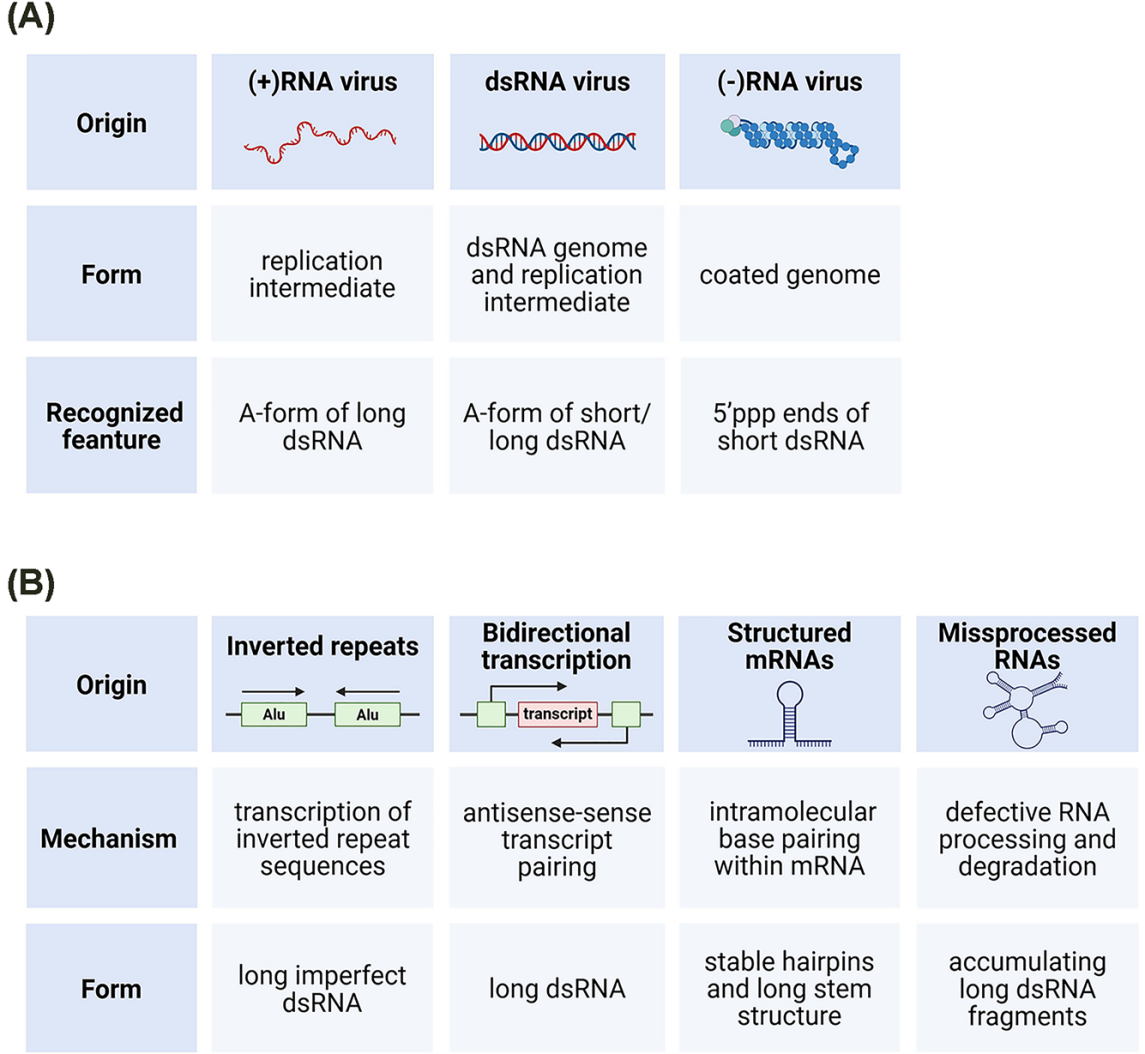
Sources of dsRNA in human cells dsRNA encountered by innate immune sensors originates from both (**A**) exogenous and (**B**) endogenous sources. (A) Viral infection introduces dsRNA into the cytoplasm through multiple routes. Positive-sense (+) RNA viruses and dsRNA viruses generate long accessible dsRNA replication intermediates, whereas dsRNA viruses additionally introduce genomic dsRNA. In contrast, most negative-sense (−) RNA viruses shield long duplexes within nucleoprotein complexes and instead expose short duplex regions with 5′-triphosphorylated termini that serve as potent ligands for cytosolic RNA sensors. (B) Endogenous dsRNA arises from intrinsic features of the transcriptome, including pairing of inverted repeat elements (e.g., Alu sequences), bidirectional transcription of overlapping loci, stable secondary structures within mRNAs, and accumulation of misprocessed or aberrant RNAs. Under homeostatic conditions, these duplexes are edited, retained, or resolved, but failure of these safeguards allows endogenous dsRNA to access innate immune sensors. Figure was created using Biorender.com.

**Figure 2:**
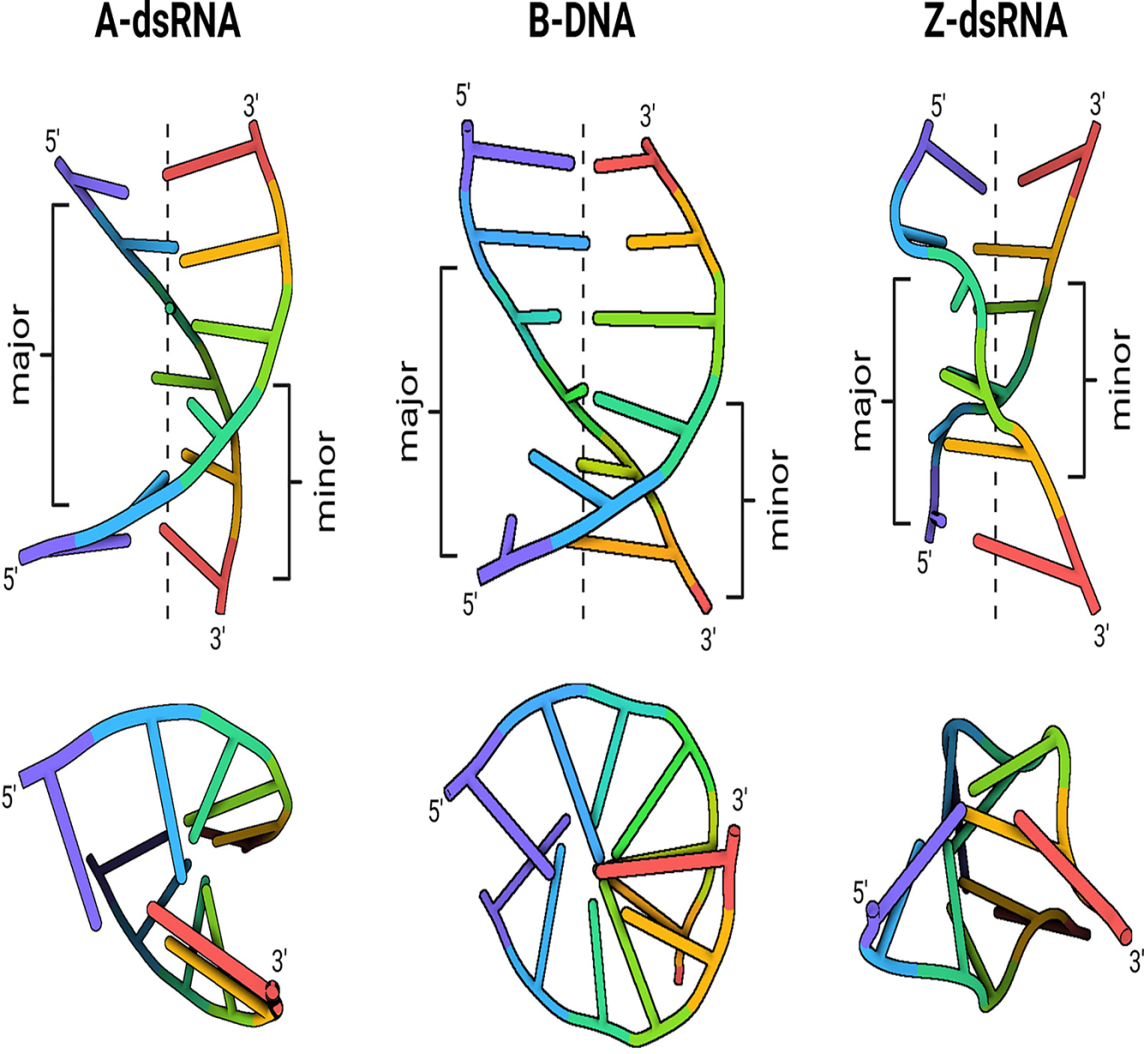
Geometry of RNA duplexes Side and top views of nucleic acid duplexes illustrating geometries relevant for innate immune recognition: right-handed A-form dsRNA, right-handed B-form DNA, and left-handed Z-form dsRNA. The structures highlight differences in helical handedness, groove dimensions, and phosphate backbone organisation that underlie selective recognition by cellular sensors. Duplex models were generated from experimentally determined atomic structures deposited in the Protein Data Bank (PDB; 9I8B, 4C64, 2GXB). Figure was created using Biorender.com.

Human cells continuously monitor the structure, localisation, and biochemical state of their RNA molecules to preserve homeostasis and mount effective defences against viral infection. Among many molecular patterns encountered in the cytoplasm, double-stranded RNA (dsRNA) is one of the most potent and evolutionarily conserved indicators of danger. Viral genomes, replication intermediates, and structured transcripts frequently contain dsRNA, although the form, length, and accessibility of these duplexes vary widely across virus families [1,2] (Figure 1A). In dsRNA viruses, long A-form duplexes are an inherent feature of the viral genome itself, while additional dsRNA species arise during transcription and replication [3] (Figures 1A and 2). Many positive-sense RNA viruses generate abundant and accessible long replication intermediates that readily adopt stable A-form helices (Figure 1A). In contrast, most negative-sense RNA viruses tightly encapsidate their genomes and antigenomes within nucleoprotein complexes, thereby limiting exposure of long naked duplex RNA. Instead, these viruses predominantly present short duplex regions with 5′-triphosphorylated termini, which serve as a potent ligands for cytoplasmic RNA sensors [4,5] (Figure 1A). Despite these differences, the unifying principle is that viral infection introduces RNA structures—whether extended helices or short duplexes bearing triphosphorylated ends—that deviate sharply from the architecture of normal cellular RNA. This highlights dsRNA as a shared molecular signature across otherwise diverse viral replication strategies.

Also endogenous dsRNA arises in the cell and originates from intrinsic features of the transcriptome, including pairing of inverted repeat elements such as Alu sequences, bidirectional transcription of overlapping loci, formation of stable secondary structures within mRNAs, and the accumulation of misprocessed or aberrant transcripts [6] (Figure 1B). Under homeostatic conditions, these duplexes are shielded from innate immune sensors through multiple layers of regulation, including RNA editing, nuclear retention, RNA processing, epigenetic silencing of repetitive elements, and controlled RNA decay. When these safeguards fail, endogenous dsRNA can access cytosolic or endosomal sensors and trigger antiviral signalling.

A defining feature of innate recognition is the ability of sensors to identify the A-form geometry of dsRNA—a right-handed helix characterised by a deep and narrow major groove, a wide and shallow minor groove, and a highly ordered, densely negative phosphate backbone [7,8] (Figure 2). Unlike the flexible B-form helix of DNA, this configuration enables sequence-independent detection and can accommodate a diverse array of viral RNAs. Because long and stable dsRNA is normally scarce in the cytoplasm, its appearance alarms cell, signalling that endogenous RNA metabolism has gone awry or viral replication is ongoing. Although A-form dsRNA is right-handed, RNA can also transiently adopt alternative conformations, including left-handed Z-RNA (Figure 2), which is selectively recognised by specific host proteins [9].

Detection of dsRNA triggers a broad reconfiguration of cellular physiology: induction of interferons and inflammatory cytokines, suppression of translation, degradation of viral and host RNA, remodelling of cytoplasmic condensates, and restructuring of the transcriptome [1,2]. The sensors that drive these responses operate primarily through structural recognition, detecting the geometric and electrostatic hallmarks of A-form duplex RNA rather than specific sequences [7,8]. This strategy allows broad and flexible surveillance while limiting opportunities for viral evasion.

**Figure 3:**
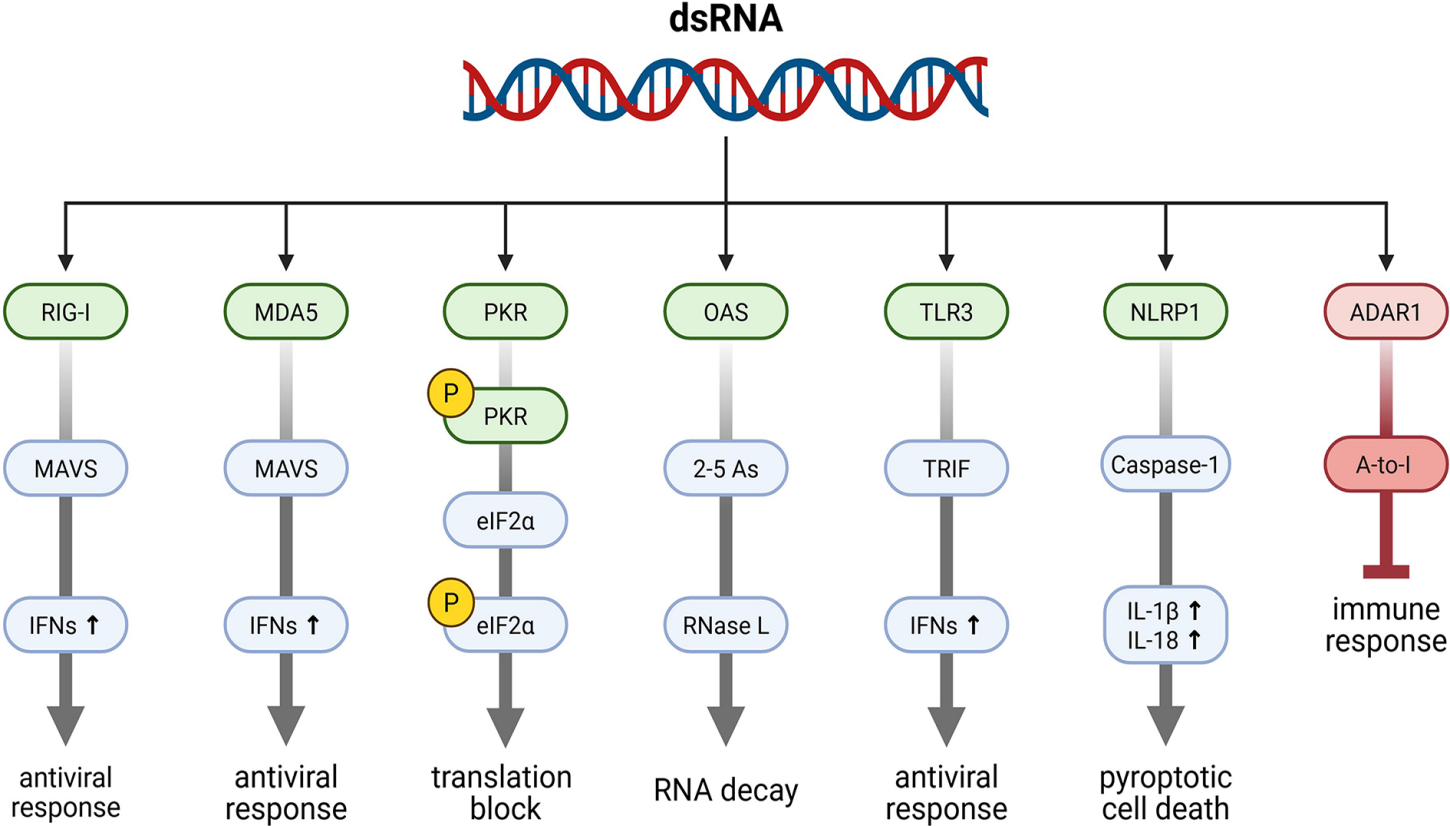
Cellular consequences of dsRNA sensing Recognition of dsRNA activates multiple innate immune pathways that collectively reprogram cellular physiology. The cytosolic sensors RIG-I and MDA5 initiate MAVS-dependent induction of type I and III interferons (IFNs) and inflammatory gene expression, with RIG-I often acting as an early detector of viral RNA termini and MDA5 contributing predominantly during sustained replication when long dsRNA intermediates accumulate. PKR links dsRNA detection to translational repression through phosphorylation of eIF2α and engagement of the integrated stress response. The OAS/RNase L pathway converts dsRNA recognition into widespread RNA degradation. Endosomal TLR3 detects internalised dsRNA and activates TRIF-dependent interferon and inflammatory signalling. In parallel, the inflammasome sensor NLRP1 can be engaged by dsRNA directly or indirectly through ribotoxic stress, leading to caspase-1 activation, cytokine maturation, and pyroptotic cell death. Counterbalancing these responses, ADAR1 edits endogenous dsRNA to prevent inappropriate immune activation and maintain self-tolerance. Figure was created using Biorender.com.

Multiple specialised systems cooperate to detect dsRNA (Figure 3). In the cytoplasm, the RIG-I-like receptors (RIG-I, MDA5, and LGP2) initiate interferon signalling [10,11]; protein kinase R (PKR) links dsRNA to global translation control [12]; and the oligoadenylate synthase (OAS)/RNase L pathway enzymatically degrades RNA [13,14]. Counterbalancing these responses, adenosine deaminase acting on RNA 1 (ADAR1) edits endogenous dsRNA in both the nucleus and cytoplasm, altering RNA sequence and structure through adenosine-to-inosine conversion to prevent inappropriate activation of cytosolic sensors and maintain self-tolerance [15]. The endosomal receptor Toll-like receptor 3 (TLR3) monitors dsRNA that enters cells through uptake of viruses, phagocytosed debris, or extracellular RNA released during tissue injury [16]. Recently also the inflammasome sensor NLR family pyrin domain containing 1 (NLRP1) has been implicated in detecting dsRNA, either through direct binding or through ribotoxic stress pathways that respond to structured viral RNA, linking dsRNA exposure to caspase-1 activation and pyroptosis [17,18]. Acting together, these systems provide multilayered, compartment-specific, and tightly regulated antiviral defence.

This mini-review synthesises mechanistic insights into how human cells detect dsRNA, how individual sensors differ in their modes of recognition and signalling, and how chemical modifications shape the immunogenicity of RNA duplexes. By examining these pathways together, we highlight how structural recognition, biochemical editing, and compartmental context allow cells to distinguish viral dsRNA from modified self RNA and to balance antiviral defence with immune tolerance.

## Key factors in dsRNA recognition and their mode of action

### PKR: translational control and integration of dsRNA with the stress response

PKR is a central effector that couples dsRNA detection to global inhibition of protein synthesis [12]. Although transcription of this kinase is strongly induced by interferons, PKR is expressed at basal levels in most types of cells [19]. Its N-terminal region contains two tandem dsRNA-binding domains (dsRBDs) that bind the minor groove of A-form duplex RNA through shape and charge complementarity [20,21]. This recognition is sequence-independent and relies mainly on the structural regularity and rigidity of the helix.

**Figure 4:**
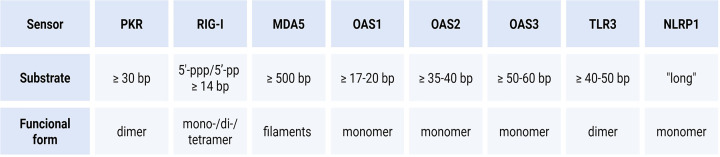
dsRNA length requirements and oligomerisation states of innate immune receptors Comparative summary of cytosolic and endosomal dsRNA sensors highlighting the minimal duplex length required for productive activation and the oligomeric state associated with signalling competence. The table illustrates that dsRNA sensors differ markedly in how they translate RNA structure into activation: RIG-I recognises short blunt-ended duplexes with 5′-triphosphorylated (5′-ppp) or diphosphorylated (5′-pp) termini and can signal as a monomer or small oligomer; PKR requires longer duplexes that support cooperative dimerisation and multimer stabilisation; MDA5 is activated only on long uninterrupted helices that permit filament assembly; OAS family members exhibit a length-sensing hierarchy (OAS1, OAS2, OAS3) that reflects the number of dsRNA-binding interfaces; TLR3 requires sufficiently long duplexes to bridge two ectodomains and stabilise receptor dimerisation; and NLRP1 responds to long dsRNA either through direct interaction with extended helices or indirectly via ribotoxic stress mechanisms that promote inflammasome assembly. Together, these properties define distinct activation thresholds and shape how individual dsRNA sensors are engaged depending on RNA structure and cellular context. Figure was created using Biorender.com.

Activation of PKR requires duplexes of at least ∼30 base pairs (bp) (Figure 4), which provide sufficient surface to recruit two protein molecules in close proximity [22,23]. Binding of dsRNA promotes protein dimerisation and autophosphorylation of the residues within the activation loop, converting enzyme into an active kinase [24]. Importantly, PKR activation strongly depends on ligand length: longer uninterrupted dsRNA helices can accommodate multiple PKR dimers along their length, enabling cooperative stabilisation of kinase–kinase interaction and producing more robust and sustained signalling [23]. Extended dsRNA replication intermediates produced by many RNA viruses are therefore exceptionally potent PKR agonists [1,2].

Activated PKR phosphorylates the α-subunit of eukaryotic initiation factor 2 (eIF2α) at Ser51, preventing eIF2B-mediated exchange of GDP for GTP on eIF2 and sharply reducing formation of the ternary translation initiation complex composed of eIF2-GTP-Met-tRNAiMet [12,24]. This results in rapid global translational shutdown, while allowing selective translation of upstream open reading frame-regulated mRNAs such as ATF4 that initiate stress-adaptive transcriptional programmes [25]. PKR activation is frequently accompanied by assembly of stress granules (SGs), cytoplasmic ribonucleoprotein condensates that form downstream of eIF2α phosphorylation [26,27]. Although SGs have been proposed to concentrate innate immune factors, recent studies indicate that their formation is not required for PKR, RLR, or OAS/RNase L pathway activation. Rather than acting as primary signalling platforms, SGs likely reflect a downstream consequence of translational arrest and may modulate RNA metabolism without being essential for antiviral signalling [28,29]. Through these mechanisms, PKR integrates structural recognition of dsRNA with translational repression, engagement of integrated stress response (ISR), and antiviral defence [30,31].

Recent work by Ahmad et al. has further refined our understanding of PKR self versus non-self discrimination. The dsRNA-binding protein PACT has been shown to prevent aberrant activation of PKR by endogenous dsRNA without simply sequestering RNA ligands, suggesting a surveillance mechanism that limits inappropriate activation while preserving responsiveness to viral RNA [32]. These findings underscore that PKR activation is shaped not only by RNA structure but also by regulatory protein interactions that help enforce immune tolerance.

### The oligoadenylate synthase/RNase L system: enzymatic conversion of dsRNA detection into RNA decay

The OAS/RNase L axis provides one of the most direct and rapid antiviral defences in human cells, converting dsRNA detection into immediate biochemical activity. Members of the OAS family are enzymes that share a conserved nucleotidyl-transferase (NTase) core that is catalytically inactive until the protein binds RNA duplex of A-form geometry [14]. Engagement of dsRNA induces an allosteric rearrangement of the NTase domain that enables synthesis of 2′-5′-linked oligoadenylates (2-5A), the obligate second messenger that activates RNase L [13] (Figure 3).

Although OAS1, OAS2, and OAS3 use the same catalytic mechanism, they differ in their dsRNA length requirements due to the number of dsRNA-binding modules they contain [33,34] (Figure 4). OAS1 harbours a single dsRNA-binding interface and responds to short helices of ∼17–20 bp. OAS2 contains two tandem dsRBDs and requires ∼35–40 bp. OAS3, which possesses three RNA-binding domains, is specialised in detecting long uninterrupted duplexes of ≥50–60 bp length. This architecture forms a natural length-sensing hierarchy, with OAS3 acting as the dominant early detector of viral replication intermediates [35].

Notably, OAS3 is expressed at appreciable basal levels in many types of human cells [35,36]. Unlike OAS1 and OAS2, which rely more on interferon-mediated transcriptional induction to reach effective concentrations, OAS3 is often present at levels sufficient to activate RNase L immediately upon encountering long dsRNA. Thus, the OAS3/RNase L system can function as an early cytosolic sentinel that responds before interferon-driven antiviral transcriptional programmes are fully engaged.

Once activated, RNase L rapidly reshapes the cytoplasmic RNA landscape by cleaving single-stranded regions of RNA at preferred UN∧N motifs. This reaction generates characteristic RNA fragments with a 5′ hydroxyl group and a 2′,3′-cyclic phosphate at the 3′ end, reflecting the metal-independent catalytic mechanism of RNase L [37–39]. These cleavage products are rapidly processed by cellular exonucleases such as XRN1 [40–42]. While early models proposed that RNase L-generated fragments could amplify antiviral signalling [43,44], more recent studies indicate that RNase L activity often constrains excessive type I interferon responses, underscoring its role in shaping rather than simply amplifying innate immunity [45,46].

Despite its broad activity, RNase L does not cleave all transcripts uniformly. Highly translated transcripts and GC-rich mRNAs are relatively protected, whereas AU-rich or poorly translated transcripts are rapidly degraded. Notably, many interferon-stimulated mRNAs persist during RNase L activation and subsequently dominate translation once the bulk cytoplasmic RNA pool has been cleared [47,48].

Beyond RNA cleavage, emerging evidence indicates that the OAS/RNase L axis influences additional layers of gene regulation. Recent studies suggest roles for RNase L in modulating host and viral protein synthesis through effects on nuclear RNA biogenesis, mRNA export, and RNA processing [45,49,50]. These functions further position RNase L as a global regulator of RNA homeostasis rather than solely a degradative effector.

Through these activities, the OAS/RNase L pathway serves both as an antiviral effector and an early translatome-reprogramming mechanism, which reshapes the RNA landscape before interferon-driven transcription fully unfolds.

### RIG-I-like receptors: raising the alarm upon diverse dsRNA feature recognition

The RIG-I-like receptors (RLRs)—RIG-I, MDA5, and LGP2—constitute the primary cytosolic signalling pathway for detecting viral dsRNA and initiating type I and III interferon responses [10,11] (Figure 3). Although all three receptors share a DExD/H-box helicase core and C-terminal dsRBD, each receptor recognises different RNA structures and employs distinct signalling strategy, enabling comprehensive detection of diverse viral replication intermediates.

#### RIG-I (Retinoic acid-inducible gene I)

RIG-I specialises in detecting short dsRNA segments with blunt ends bearing 5′-triphosphate (5′-ppp) or diphosphate (5′-pp) groups—molecular signature common in viral RNAs but almost absent from mature host transcripts [4,51] (Figure 4). In resting cells, RIG-I adopts an autoinhibited conformation in which its N-terminal caspase recruitment domains (CARDs) dock against the helicase core [52]. Binding to a 5′-ppp blunt end positions the C-terminal domain on the terminal base pairs and induces ATP-dependent closure of the helicase domains, releasing the CARDs and enabling interaction with the mitochondrial antiviral-signalling (MAVS) adaptor protein [53,54].

RIG-I is unusually flexible in its oligomeric requirements for signalling. It can activate antiviral responses as a monomer, dimer, or tetramer, with the tetrameric ‘lockwasher’ assembly representing the most stable and potent signalling conformation [55,56]. Monomeric RIG-I relies heavily on K63-linked polyubiquitination mediated by TRIM25 and RIPLET, which stabilises exposed CARDs and supports MAVS engagement [55,57–59]. In contrast, tetrameric RIG-I relies less on ubiquitin scaffolding because protein–protein interfaces within the oligomer itself stabilise the signalling-competent CARDs arrangement [55]. This flexibility allows RIG-I to efficiently initiate signalling even when the level of sensor expression is relatively low, making RIG-I a highly sensitive detector of viral RNA termini.

#### MDA5 (Melanoma differentiation associated gene 5)

MDA5 recognises long, uninterrupted dsRNA helices typical of picornaviruses and other viruses that generate extended replication intermediates [60–63] (Figure 4). Unlike RIG-I, which binds ends of RNA, MDA5 engages internal regions of dsRNA and assembles cooperative, ATP-dependent filaments along the duplex [63–65]. Filament formation serves both as a detection mechanism and as a proofreading step: mismatches, bulges, or local irregularities interrupt filament propagation, ensuring selective recognition of viral dsRNA while excluding heterogeneous cellular endogenous duplexes.

Effective MDA5 signalling requires dense filament assembly and sufficient receptor abundance [63–65]. In many experimental systems, basal MDA5 expression is relatively low, and is markedly increased upon interferon stimulation [66]. This observation has led to the view that MDA5 often functions as a secondary or amplifying sensor during later stages of infection [10,67]. Importantly, however, there is no intrinsic mechanistic requirement for prior RIG-I activation. If basal MDA5 levels are sufficiently high, long dsRNA can, in principle, initiate signalling independently. Thus, MDA5 activation depends not only on ligand structure but also on the cell-type-specific receptor abundance.

Although MDA5 is often described as a sensor of long uninterrupted viral dsRNA, genetic and pharmacological studies demonstrate that it can also respond to endogenous RNA under conditions of dysregulated RNA metabolism, such as ADAR1 deficiency, epigenetic de-repression of transposable elements, or splicing inhibition [68–70]. Notably, many endogenous editing substrates are shorter and structurally imperfect relative to classical viral replication intermediates, and the precise identity of physiologically relevant endogenous MDA5 ligands remains an area of active investigation [6]. Proposed candidates include inverted-repeat Alu duplexes, cis-natural antisense transcripts, and mitochondrial dsRNA species.

Like RIG-I, MDA5 requires K63-linked ubiquitination—partially mediated by TRIM65 [71,72]—for efficient signalling [73,74]. However, the mechanistic role of ubiquitination differs between two receptors. For MDA5 ubiquitination primarily stabilises CARD clustering within higher-order filament assemblies rather than driving CARD exposure. In contrast, ubiquitin scaffolding plays a more pronounced role in RIG-I signalling, where it is critical for stabilising exposed CARDs in monomeric or small oligomeric states that nucleate MAVS filaments [55,57–59]. Thus, while ubiquitination is essential for both receptors, MDA5 signalling is dominated by filament assembly and receptor abundance, whereas RIG-I signalling is more sensitive to terminal RNA features and ubiquitin-mediated regulation.

#### LGP2 (Laboratory of genetics and physiology 2)

LGP2 lacks CARD domains and therefore is unable to signal autonomously, but it plays important regulatory roles within RLR pathway [10,11]. LGP2 enhances MDA5 sensitivity by stabilising initial RNA binding and filament nucleation [75]. Conversely, by binding dsRNA ends, LGP2 can compete with RIG-I and dampen its activation, especially during later stages of infection when LGP2 expression is elevated [76]. Because LGP2 is itself induced by interferons, it participates in both feed-forward enhancement of MDA5 responses and negative feedback regulation of RIG-I signalling.

Recent mechanistic studies further indicate that LGP2 can regulate MDA5 filament dynamics through ATP-dependent one-dimensional movement along dsRNA, thereby suppressing spontaneous filament assembly in the absence of infection while promoting appropriate activation during viral challenge [77]. This dual role highlights LGP2 as a dynamic modulator of MDA5 homeostasis rather than merely a static accessory factor.

#### Functional hierarchy

The relationship between RIG-I and MDA5 usually is described as hierarchical, where RIG-I acts as an early sensor and MDA5 contributes predominantly at later stages of infection. This view is supported by the strong immunogenicity of 5′-triphosphorylation of RNA, which determines recognition of also long duplex which length would otherwise favour MDA5 engagement [36]. However, such a hierarchy should not be regarded as universal.

Basal expression levels of RIG-I and MDA5 vary across cell types and are constrained by the need to tolerate endogenous dsRNA without triggering immunity. These levels therefore reflect the highest sensor abundance compatible with self-tolerance, a balance maintained in part by ADAR1-mediated RNA editing [15]. Consequently, the relative contribution of RIG-I and MDA5 to antiviral sensing is context-dependent rather than universally fixed.

Either receptor may act as the primary initiator of antiviral signalling depending on cellular context. In some conditions, basal MDA5 levels may be sufficient to respond directly to long structurally regular intermediates of viral dsRNA replication, with interferon signalling subsequently amplifying RIG-I expression. In others, RIG-I may dominate early sensing through its sensitivity to terminal RNA features. Thus, rather than a strict linear hierarchy, RIG-I and MDA5 operate as a flexible and context-dependent sensing module, with their relative contributions defined by receptor abundance, RNA structure, and cellular RNA homeostasis.

### TLR3: endosomal recognition of internalised dsRNA

TLR3 is a member of the Toll-like receptor family, whose members survey extracellular and endosomal compartments for conserved microbial structures. Among TLRs, TLR3 is uniquely specialised for sensing dsRNA, providing a complementary, compartmentalised arm of RNA surveillance that operates after viral entry, phagocytosis, or uptake of extracellular RNA released during tissue damage [16].

Structurally, TLR3 differs fundamentally from cytosolic dsRNA receptors. Rather than probing the minor groove of an A-form helix, TLR3 uses a large horseshoe-shaped ectodomain composed of leucine-rich repeats (LRRs) [78]. This LRR solenoid presents an extended, positively charged surface that interacts primarily with the negatively charged phosphate backbone of dsRNA. Because TLR3 binds dsRNA through a wide, positively charged surface on its LRR ectodomain—rather than by probing fine helix geometry—it effectively reads dsRNA as a repeating pattern of negative charge, tolerating many mismatches and modifications provided the duplex remains long and rigid.

A single dsRNA duplex bridges two TLR3 ectodomains to form a ligand-stabilised dimer; each monomer binds one side of the helix, and dimerisation juxtaposes the intracellular Toll/interleukin-1 receptor (TIR) domains [79]. These TIR domains recruit the Toll/interleukin-1 receptor domain-containing adaptor inducing interferon β (TRIF) protein, which is distinct from the myeloid differentiation primary response 88 (MyD88) pathway used by most other TLRs, and initiate downstream activation of interferon regulatory factor 3 (IRF3) and nuclear factor kappa-light-chain-enhancer of activated B cells (NF-κB) to drive type I interferon and proinflammatory cytokine expression [80–82] (Figure 3). Ligand length strongly influences activation: duplexes on the order of ∼40–50 bp are minimally stimulatory (Figure 4), whereas longer and more rigid helices promote more efficient dimerisation and signalling, consistent with the spatial requirements of two LRR solenoids [83–85].

Endosomal acidification adds another layer of specificity. TLR3 binds dsRNA most avidly in acidic environments where protonation of histidine residues stabilises receptor-ligand contacts [86]; this ensures that TLR3 responds primarily to dsRNA encountered within endocytic or phagocytic compartments rather than to cytosolic transcripts. Because TLR3 engages dsRNA mainly through electrostatic and surface contacts with the phosphate-ribose backbone, alterations that change phosphate accessibility, hydration or local charge—for example phosphorothioate backbone substitutions, ribose 2′-*O*-methylation (2′-*O*-Me), or nucleoside isomerization such as pseudouridine—can diminish receptor binding and downstream signalling by changing the electrostatic landscape and/or the duplex rigidity required to bridge two LRR ectodomains [87–91].

### ADAR1: editing dsRNA to preserve self-tolerance

ADAR1 serves as the major guardian of self-RNA integrity by editing endogenous dsRNA to prevent inappropriate activation of cytosolic dsRNA sensors [15] (Figure 3). The enzyme catalyses the deamination of adenosine to inosine (A-to-I) within RNA duplexes. Because inosine pairs with uridine to form an I:U wobble pair, editing weakens local base pairing, destabilises helix stacking, and disrupts the long uninterrupted A-form structures required for activation of MDA5 and OAS enzymes, as well as, to a lesser extent, PKR [15,28,69,92–95]. Through this action ADAR1 converts endogenous dsRNA with immunostimulatory potential into structurally imperfect helices that are poorly recognised by innate immune sensors.

Importantly, accumulating evidence suggests that most endogenous editing substrates are not thought to represent *bona fide* activating ligands for dsRNA sensors under physiological conditions. Rather, only a subset of structured RNAs—whose precise identity remains debated—appears capable of reaching activation thresholds in the absence of editing [96,97]. In addition to editing endogenous duplexes, ADAR1 can also modify viral RNA genomes and replication intermediates, although the functional consequences of such editing are highly context- and virus-specific, ranging from attenuation of viral replication to facilitation of immune evasion [98,99].

ADAR1 exists in two isoforms with complementary and non-redundant functions [100,101]. The constitutively expressed p110 isoform is predominantly nuclear, where it edits structured regions of mRNA precursors (pre-mRNAs) and nuclear dsRNAs, preventing excessive accumulation of duplex RNA during transcript processing and maturation. In contrast, the interferon-inducible p150 isoform resides mainly in the cytoplasm and is essential for maintaining immune tolerance. Cytoplasmic p150 edits long imperfect duplexes derived from inverted repeats such as Alu elements—the predominant source of endogenous dsRNA that would otherwise activate MDA5, PKR, or OAS [15,28,69,92–95]. Loss of ADAR1p150, or disruption of its RNA-binding specificity, results in chronic MDA5-driven interferon production and autoinflammatory disease, most notably Aicardi–Goutières syndrome [102].

Two isoforms of ADAR1 differ not only in localisation but in their modes of RNA recognition. Both p110 and p150 variants contain multiple dsRBDs that engage the minor groove and phosphate backbone of canonical A-form helices, allowing ADAR1 to bind long duplexes independently of their sequence. In addition, ADAR1p150 possesses an additional N-terminal Zα domain, which can bind left-handed Z-RNA [103] (Figure 2)—a transient RNA conformation favoured in GC-rich or repetitive sequences and promoted under cellular stress conditions or high transcriptional activity [104]. Recognition of Z-RNA does not replace the ability to bind A-form; rather, it expands the repertoire of substrates accessible to ADAR1p150, enabling efficient detection of self dsRNA structures that become more abundant during inflammation.

Through its coordinated isoform-specific functions and its ability to recognise both canonical A-form helices and transient Z-RNA segments, ADAR1 systematically edits endogenous dsRNA landscape. By editing self-derived duplexes before they can engage innate immune sensors, ADAR1 establishes a critical structural boundary between self and non-self dsRNA and is indispensable for maintaining immune homeostasis in human cells.

### NLRP1 and inflammasome activation: direct binding and ribotoxic stress pathways

Recent work by Bauernfried et al. has added a third dimension to dsRNA-triggered innate responses by implicating the NLRP1 inflammasome in RNA sensing [17]. Evidence supports two complementary modes by which dsRNA can engage NLRP1. In the first one, NLRP1 directly interacts via its LRR domain with long duplex RNA: long synthetic helices or extended dsRNA originating from viral infection stimulate formation of NLRP1–ASC (apoptosis-associated speck-like protein containing a CARD)-caspase-1 inflammasomes, resulting in maturation of pro-inflammatory cytokines, such as interleukin (IL) 1β and IL-18, and execution of pyroptotic cell death [17]. A second model proposes a mechanistically distinct route in which excessive dsRNA or other translation-disrupting stressors induce ribosome stalling and collision events. These ribotoxic stress signals activate the kinase ZAKα, which promotes NLRP1 functional degradation and inflammasome assembly [18]. ZAKα signalling, via downstream effectors such as p38, promotes phosphorylation and functional degradation of an N-terminal region of NLRP1, liberating its C-terminal FIIND–CARD fragment to assemble the inflammasome [18,105].

These two pathways are not mutually exclusive: their relative contribution likely depends on cell type, NLRP1 expression level, RNA length and structure, and whether the duplex directly binds to the receptor or instead induces ribosomal stress. Functionally, the duality emphasises that dsRNA exposure can channel infected or damaged cells not only into antiviral interferons and translational control programmes, but also into inflammasome-driven inflammatory and lytic responses. Integrating NLRP1 into the broader dsRNA-sensing network therefore widens the range of possible cellular outcomes and highlights another axis by which RNA structure and cellular context determine immune fate.

## Epitranscriptomic marks and chemical modifications: shaping dsRNA immunogenicity

**Figure 5:**
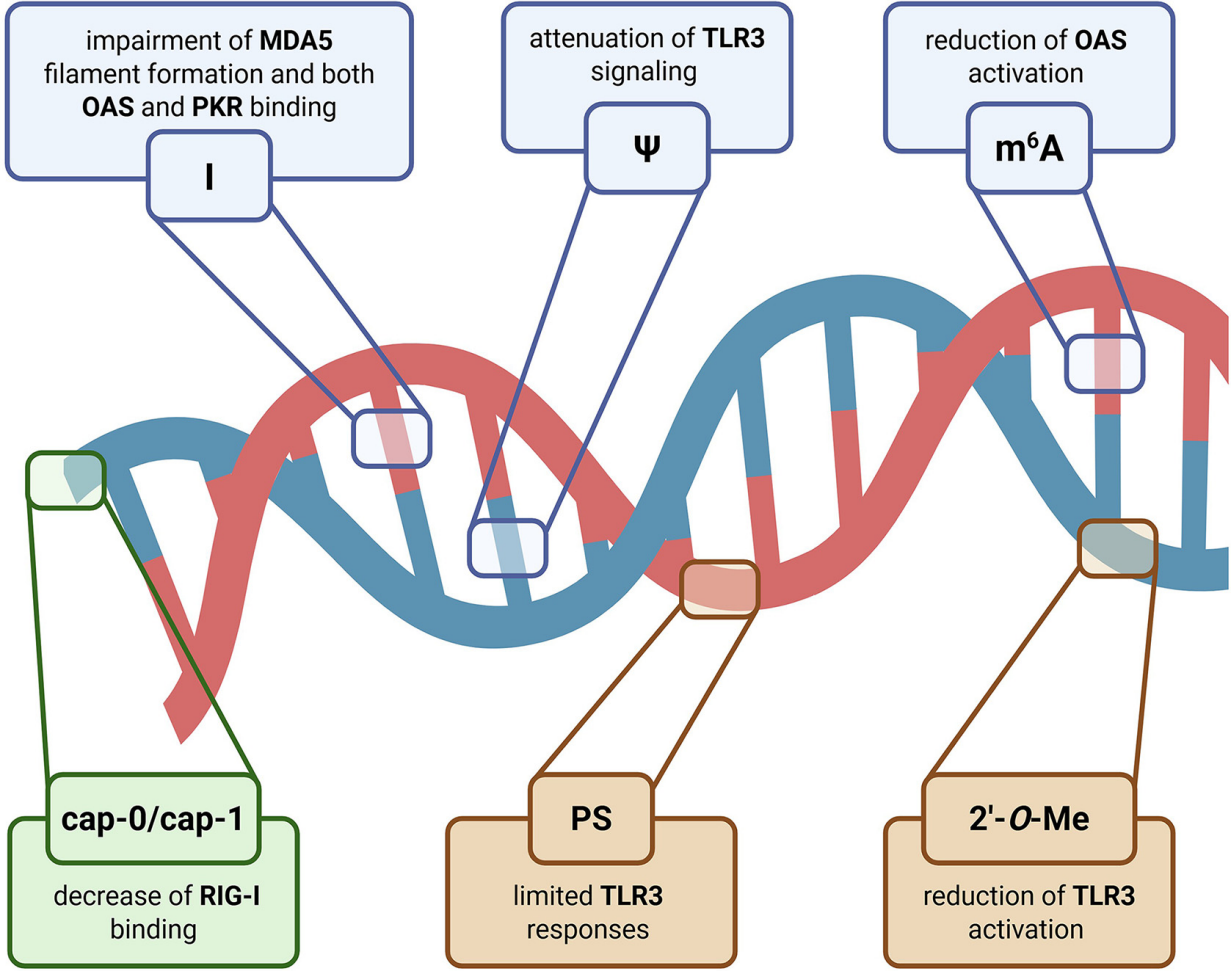
Impact of epitranscriptomics marks and chemical modifications on dsRNA immunogenicity Chemical modifications and epitranscriptomic marks modulate dsRNA immunogenicity by altering helix geometry, backbone electrostatics, hydration, and terminal structure. Internal base modifications such as *N*^6^-methyladenosine (m^6^A), pseudouridine (Ψ), and ADAR1-mediated inosine (I) introduce local structural irregularities that differentially affect sensor engagement. Ribose and backbone modifications, including 2′-*O*-methylation (2′-*O*-Me) and phosphorothioate (PS) substitution, reshape hydration shells and phosphate charge distribution. Terminal features at the 5′ end, including 5′-triphosphate, cap-0, and cap-1 structures, are decisive for receptors that interrogate RNA termini such as RIG-I. Together, these modifications reduce or prevent activation of specific dsRNA sensors and contribute to a biochemical ‘self-code’ that distinguishes endogenous RNA from viral dsRNA. Figure was created using Biorender.com.

Chemical modifications and epitranscriptomic marks critically shape how dsRNA is interpreted by innate immune sensors [106] (Figure 5). Because dsRNA recognition is largely sequence-independent and relies on detection of A-form helical geometry [7,8], the introduction of modifications that alter base pairing, stacking, backbone chemistry, or terminal structure can profoundly reshape the immunogenic potential of RNA duplexes. Importantly, these effects are not uniform across sensors; rather, each sensor displays a distinct sensitivity profile that reflects its underlying mode of RNA engagement and signal transduction.

### Internal base modifications: pseudouridine, *N*^6^-methyladenosine, and inosine

Modifications of bases in RNA body primarily affect dsRNA immunogenicity by modulating base-stacking interactions and local helical geometry. Their consequences for sensing depend on whether these changes disrupt the continuity, rigidity, or regularity of the A-form helix.

Pseudouridine (Ψ) stabilises base stacking and subtly alters hydrogen-bonding and hydration without intrinsically compromising A-form geometry [107–109]. As a result, Ψ-containing duplexes generally remain competent ligands for PKR, MDA5, and OAS enzymes as long as helix length and continuity are preserved [36,93,110–112]. These sensors tolerate Ψ unless its incorporation density or positioning induces local bending or irregularity sufficient to interfere with PKR dimerisation, MDA5 filament propagation, or OAS allosteric activation. TLR3, which relies heavily on electrostatic interactions with the phosphate backbone, is more sensitive to Ψ-associated changes in hydration and can show reduced signalling even when duplex integrity is maintained [87–89,91]. Thus, pseudouridine modulates dsRNA sensing indirectly, through structural effects rather than direct antagonism of cytosolic sensors.

*N*^6^-methyladenosine (m^6^A) weakens base stacking and slightly widens local groove geometry, introducing local flexibility into the duplex [107,113,114]. PKR shows tolerance to sparse m^6^A incorporation, which is consistent with its ability to bind imperfect helices provided that sufficient duplex length and rigidity remain [36,110,111]. In contrast, OAS enzymes are highly sensitive to m^6^A-induced distortions because catalytic activation of these enzymes requires a near-ideal A-form helix [36,112]; even subtle deviations can markedly reduce 2-5A synthesis. Genetic and cellular studies strongly support a role for m^6^A in modulating MDA5 signalling [93,115,116]; however, direct structural or single-molecule evidence demonstrating how m^6^A alters MDA5 filament assembly remains limited. Its impact on MDA5 is therefore inferred from cellular and biochemical readouts and by analogy to other helix-destabilising modifications. Effects on RIG-I are indirect and context-dependent, arising primarily when m^6^A destabilises base pairing near duplex termini and promotes end fraying [117].

A-to-I editing by ADAR1 is the most potent internal modification which shapes dsRNA immunogenicity [107]. Conversion of adenosine to inosine introduces I:U wobble pairs that weaken local base pairing and break long uninterrupted A-form helices [118,119]. These discontinuities efficiently prevent MDA5 filament formation and strongly impair OAS activation, at the same time reducing PKR dimerisation efficiency [28,69,92,110–112]. Through cumulative editing, endogenous dsRNAs are converted from potent ligands into imperfect helices that fall below activation thresholds of multiple cytosolic sensors.

### Backbone and ribose modifications: phosphorothioates and internal 2′-*O*-Me

Backbone and ribose modifications influence dsRNA sensing primarily by altering electrostatics, hydration, and conformational flexibility rather than base pairing [120]. Phosphorothioate substitution replaces a non-bridging phosphate oxygen with sulphur, reducing local negative charge density and reshaping hydration shells along the duplex [121,122]. These changes have relatively modest effects on sensors such as PKR and MDA5, which rely more on helix length and overall geometry than on fine electrostatic readout [20,23,61,64,111]. In contrast, TLR3 is particularly sensitive to phosphorothioate substitution because its ectodomain engages dsRNA largely through electrostatic complementarity with the phosphate backbone [87,89–91]; reduced charge density weakens receptor binding and diminishes signalling even when base pairing and duplex length are preserved. OAS enzymes, like PKR and MDA5, primarily respond to overall helix length and A-form integrity, and internal ribose or backbone modifications are generally tolerated unless they disrupt duplex continuity or global helical geometry [33,123,124].

Internal 2′-*O*-Me of the ribose represents a related but mechanistically distinct modification. Addition of a methyl group at the 2′-hydroxyl reduces hydrogen-bonding capacity and alters local hydration without directly disrupting Watson–Crick base pairing [125]. When present internally within a dsRNA duplex, sparse 2′-*O*-Me is likely tolerated by PKR and MDA5 as long as the overall A-form geometry and duplex continuity are maintained [21,64,126]. However, higher densities or clustered placement can be expected to introduce local rigidity and subtle groove alterations that interfere with cooperative assembly mechanisms, particularly MDA5 filament propagation.

Considering the stringent structural requirements for OAS activation, which depend on a highly regular A-form helix and precise positioning of the phosphate backbone, internal 2′-*O*-Me is predicted to be poorly tolerated by these enzymes [123,124]. Even subtle perturbations in ribose conformation or local hydration are sufficient to impair the allosteric rearrangement required for 2-5A synthesis by OAS and subsequent RNase L activation. TLR3 is likewise sensitive to internal 2′-*O*-Me, as reduced hydration and altered electrostatics along the backbone weaken the broad electrostatic interface through which the receptor engages dsRNA [85–87].

With respect to inflammasome signalling, internal 2′-*O*-Me is expected to dampen NLRP1 activation in both proposed modes. Reduced backbone accessibility and altered hydration would weaken direct binding to the LRR domain, while increased duplex stability and reduced conformational plasticity are likely to diminish ribosome stalling and ZAKα-dependent ribotoxic stress signalling. Although direct experimental data remain limited, these effects are consistent with the structural logic underlying both NLRP1 activation pathways.

### 5′-end modifications: triphosphate, cap-0, and cap-1

Chemical modifications at the 5′ end of RNA play a decisive role for sensors that interrogate duplex termini, most notably RIG-I. RIG-I recognition requires both a blunt duplex end and a negatively charged 5′-triphosphate or diphosphate, which together allow the receptor to clamp the terminal base pair and close its helicase domains [4,51].

Host mRNAs evade this recognition through capping [127]. Cap-0 structures, consisting of an *N*^7^-methylguanosine (N^7^MeG) linked via a 5′–5′ triphosphate bridge, partially reduce RIG-I binding, but on their own are insufficient to fully abrogate recognition [128]. Addition of 2′-*O*-Me at the first transcribed nucleotide (cap-1) acts synergistically with N7MeG to strongly suppress RIG-I engagement by sterically and electrostatically interfering with terminal recognition. This explains why mature host transcripts are effectively invisible to RIG-I despite transient exposure of phosphorylated 5′ ends during RNA biogenesis.

Importantly, endogenous RNAs can also activate RIG-I under specific conditions. For example, certain RNA polymerase III transcripts retain 5′-triphosphate ends and rely on shielding by the RNA-binding proteins which prevents inappropriate RIG-I activation [129,130]. Thus, terminal modification alone does not lead to assigning transcript as non-self.

Internal destabilisation of the duplex immediately adjacent to the 5′ end further reduces RIG-I engagement by promoting end fraying or preventing proper helicase closure, although systematic analyses of this effect remain limited [117]. MDA5 and OAS enzymes are largely insensitive to 5′-end chemistry, reflecting their reliance on internal helix features rather than terminal structures. TLR3 similarly does not interrogate RNA ends and is only affected by 5′ modifications if they influence the length or rigidity of the duplex.

## Consequences of dsRNA recognition

Recognition of dsRNA mobilises an integrated and multi-layered antiviral programme [1,2]. Engagement of RIG-I and MDA5 activates the mitochondrial adaptor MAVS, driving induction of type I and III interferons and an array of interferon-stimulated genes [10,11]. PKR phosphorylates eIF2α, enforcing global translational arrest and engaging the ISR [12,24], while the OAS/RNase L system degrades viral and host RNAs, rapidly reshaping the translatome [13,14]. In parallel, endosomal detection of dsRNA by TLR3 triggers TRIF-dependent interferon and proinflammatory signalling [80–82]. ADAR1 does not induce antiviral response but edits endogenous dsRNA to instead prevent inappropriate activation of cytosolic sensing pathways and to maintain self-tolerance [15]. When engaged, the inflammasome sensor NLRP1 links dsRNA exposure to caspase-1 activation, IL-1β and IL-18 maturation, and pyroptotic cell death [17,18].

These signalling events often coincide with remodelling of cytoplasmic architecture, including SGs and RNase L-induced bodies [131]. However, ablation of core SG components does not abolish PKR, RLR-MAVS, or OAS/RNase L activation, indicating that such condensates are downstream manifestations of pathway engagement rather than primary determinants of signalling output [28,29]. SGs have been reported to contain RNA-binding proteins and signalling components; however, their precise functional contribution to antiviral signalling remains debated [28]. Although early studies proposed that RNase L-generated RNA fragments could amplify antiviral signalling [43,44], more recent evidence suggests that these fragments are rapidly degraded and that RNase L activity frequently limits or reshapes interferon responses rather than acting as a direct secondary signalling amplifier [45,46].

## Integrated view

Human cells interpret dsRNA as a shared signal of viral infection and endogenous RNA dysregulation. A network of sensors—PKR, OAS enzymes, RIG-I, MDA5, LGP2, TLR3, and NLRP1—translates the geometry, length, and chemistry of RNA duplexes into diverse outcomes, ranging from translational shutdown and RNA decay to interferon production and inflammatory cell death. These pathways act across compartments and timescales, ensuring both rapid detection of infection and reinforcement of antiviral defences as viral replication progresses.

Counterbalancing the performance of these sensing mechanisms, ADAR1 reshapes the endogenous dsRNA landscape. Through A-to-I editing, ADAR1 prevents self-RNA from engaging innate immune receptors and thereby preserves immune tolerance. Epitranscriptomic marks further refine this discrimination by modulating duplex stability, end chemistry, and backbone properties, collectively forming an immunological ‘self-code’ that determines which sensors are engaged and to what extent.

Disruption of this precisely tuned system—whether through altered RNA modification, defective editing, or aberrant sensor activation—would result in impaired antiviral immunity, chronic inflammation, or autoinflammatory disease. Defining the molecular rules that govern dsRNA recognition and sensor-specific outputs therefore remains essential for understanding innate immune regulation and for developing therapeutic strategies that selectively amplify or restrain these pathways in infection, autoimmunity, and cancer.

## Perspectives

- dsRNA sensing lies at the core of antiviral immunity and self versus non-self discrimination, and its dysregulation contributes to diverse human diseases ranging from viral pathogenesis to autoinflammatory syndromes.
- Current models emphasise that innate immune recognition emerges from an integrated network of cytosolic, endosomal, and editing-based mechanisms that detect dsRNA through its A-form geometry and coordinate translational arrest, RNA decay, interferon signalling, and inflammasome activation.
- A central future challenge is to dissect the mechanistic individuality of each sensor—clarifying how its activity is regulated, what its specific biochemical outputs are, and how these distinct contributions combine to shape the overall innate response in different cellular contexts.

## Open Access

Open access for this article is provided via our Subscribe to Open programme, made possible through the support of our subscribing institutions. Learn more here: https://portlandpress.com/pages/open_access_options_and_prices#conditional.

## Abbreviations

2-5A: 2′-5′-linked oligoadenylates
2′-*O*-Me: 2′-*O*-methylation
5′-pp: 5′-diphosphorylated
5′-ppp: 5′-triphosphorylated
A-to-I: deamination of adenosine to inosine
ADAR1: adenosine deaminase acting on RNA 1
ASC: apoptosis-associated speck-like protein containing a CARD
bp: base pairs
CARD: caspase recruitment domain
dsRBD: dsRNA-binding domain
dsRNA: double-stranded RNA
eIF2: eukaryotic initiation factor 2
IFN: interferon
IL: interleukin
IRF3: interferon regulatory factor 3
ISR: integrated stress response
LGP2: laboratory of genetics and physiology 2
LRR: leucine-rich repeat
m^6^A: *N*^6^-methyladenosine
MAVS: mitochondrial antiviral-signallin
MDA5: melanoma differentiation associated gene 5
MyD88: myeloid differentiation primary response 88
N^7^MeG: *N*^7^-methylguanosine
NF-κB: nuclear factor kappa-light-chain-enhancer of activated B cells
NLRP1: NLR family pyrin domain containing 1
NTase: nucleotidyl-transferase
OAS: oligoadenylate synthase
PKR: protein kinase R
PS: phosphorothioate
RIG-I: retinoic acid-inducible gene I
SG: stress granule
TIR: Toll/interleukin-1 receptor
TLR: Toll-like receptor
TRIF: Toll/interleukin-1 receptor domain-containing adaptor inducing interferon β
Ψ: pseudouridine
